# Impact of left atrial appendage fibrosis on atrial fibrillation in patients following coronary bypass surgery

**DOI:** 10.1002/clc.23884

**Published:** 2022-07-21

**Authors:** Jan Eckstein, André Renner, Armin Zittermann, Thomas Fink, Christian Sohns, Karsten Niehaus, Hanna Bednarz, Judith Martha Neumann, Misagh Piran, Udo Kellner, Jan Gummert

**Affiliations:** ^1^ Department for Radiology, Nuclear Medicine and Molecular Imaging, Herz‐ und Diabeteszentrum Nordrhein‐Westfalen Ruhr‐Universität Bochum Bad Oeynhausen Germany; ^2^ Clinic for Cardiovascular and Thoracic Surgery, Herz‐ und Diabeteszentrum Nordrhein‐Westfalen Ruhr‐University Bochum Bad Oeynhausen Germany; ^3^ Clinic for Electrophysiology, Herz‐ und Diabeteszentrum Nordrhein‐Westfalen Ruhr‐University Bochum Bad Oeynhausen Germany; ^4^ Proteome and Metabolome Research, Center for Biotechnology (CeBiTec), Faculty of Biology Bielefeld University, NRW Bielefeld Germany; ^5^ Medical School OWL, AG1: Sustainable Environmental Health Sciences Bielefeld University, NRW Bielefeld Germany; ^6^ Institute for Pathology and Molecular Pathology, Johannes Wesling Klinikum Ruhr‐University Bochum Minden Germany

**Keywords:** atrial fibrillation, fibrosis, left atrial appendage

## Abstract

**Objectives:**

We aimed to assess the relationship of left atrial appendage (LAA) fibrosis with atrial fibrillation (AF) and postoperative events in patients receiving coronary artery bypass graft surgery (CABG).

**Background:**

Increased atrial fibrosis has been associated with AF and worse outcome following catheter ablation. Only limited data exists focusing on the impact of LAA fibrosis on AF after CABG.

**Methods:**

LAA tissue from 164 CABG‐patients was stained with Masson‐Goldner trichrome. The histological landscape was scanned and segmented into superpixels for software analysis (QuPath). A classification algorithm was extensively trained to detect fibrotic superpixels for quantification. In 43 propensity score matched pairs with AF or sinus rhythm (SR), LAA fibrosis was compared. Moreover, subgroups of mitral valve regurgitation (MR) were analyzed as follows: SR, SR + MR, AF and AF + MR. The predictive value of LAA fibrosis postoperative stroke, postoperative AF and mortality was assessed.

**Results:**

Fibrotic remodeling (%) showed no significant difference for the total cohort between the SR and AF group (SR: 30.8 ± 11.4% and AF: 33.8 ± 16.0%, respectively, *p* = .32). However, significant fibrotic remodeling was observed for SR and AF subgroups (SR: 27.2 ± 12.2% vs. AF: 35.3 ± 13.7%; respectively, *p* = .049) and between SR and SR + MR subgroups (SR: 27.2 ± 12.2% vs. SR + MR: 34.9 ± 9.1%, respectively, *p* = .027). LAA fibrosis was not significantly associated with postoperative stroke, postoperative AF or overall mortality (all *p* > .05).

**Conclusion:**

LAA fibrosis may contribute to an individual arrhythmia substrate for AF in patients with AF but also in those with SR and coincidence of MR. LAA fibrosis was not found to be predictive for clinical events in patients after CABG.

## Graphical abstract

Intraoperatively resected left atrial appendages (LAA) from 164 coronary artery bypass graft patients formed two compatible groups of 43 patients with and without AF after propensity score matching. Interstitial fibrosis of LAA stained with Masson‐Goldner trichrome was quantified by software. Fibrotic differences were detected after subgrouping considering mitral valve regurgitation. LAA fibrosis showed no correlation with hard clinical end points.

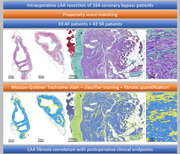

**Abbreviations**
AF: atrial fibrillation
AUC: area under the curve
BMI: body mass index
CABG: coronary artery bypass graft
LA: left atrium
LAA: left atrial appendage
MR: mitral valve regurgitation
NYHA: New York Heart Association
PS: propensity score
ROC: receiver operating characteristic
SR: sinus rhythm

## 1 INTRODUCTION

Myocardial fibrosis of the left atrium (LA) is increasingly recognized as an arrhythmogenic substrate for atrial fibrillation (AF).^1^, ^2^, ^3^, ^4^ The accumulation of fibrosis in the LA has been classified as a prognostic biomarker with regard to the risk of AF relapse after ablation.^4^, ^5^ Further histological studies have shown that the coexistence of AF and mitral valve regurgitation (MR) promote cardiac fibrotic remodeling,^1^, ^2^ exacerbating the course of disease progression.^6^, ^7^ However, the role of myocardial fibrosis in the left atrial appendage (LAA) in association with AF remains unclear.

Recent interventional approaches have shown a reduction in the rate of AF relapse when including the LAA as a therapeutic target in addition to pulmonary vein isolation.^8^, ^9^, ^10^, ^11^ Additionally, a recent multicenter study demonstrated that among 2379 patients, those receiving LAA‐occlusion (4.8%) present a significantly lowered postoperative stroke incidence compared to patients without LAA‐occlusion (7.0%).^12^ Both stroke risk and AF relapse rate are important therapeutic indications of the LAA. Structural fibrotic alterations of the LAA may substantiate why therapeutic inclusion of the LAA should be considered.

Magnetic resonance imaging studies observed increased LA fibrotic remodeling when structural heart disease and AF coexist.^13^ Therefore, it is plausible that LAA fibrotic remodeling for patients with coronary artery bypass graft surgery (CABG) is enhanced. In turn, increased LAA fibrosis may act as an arrhythmogenic substrate and is thus of potential prognostic and therapeutic importance. In this study, digital pathology was utilized to analyze the value of LAA fibrosis in association with AF for CABG patients along with follow up data regarding postoperative mortality, AF, and stroke.

## 2 MATERIALS AND METHODS

### 2.1 Subject selection

**Figure 1:**
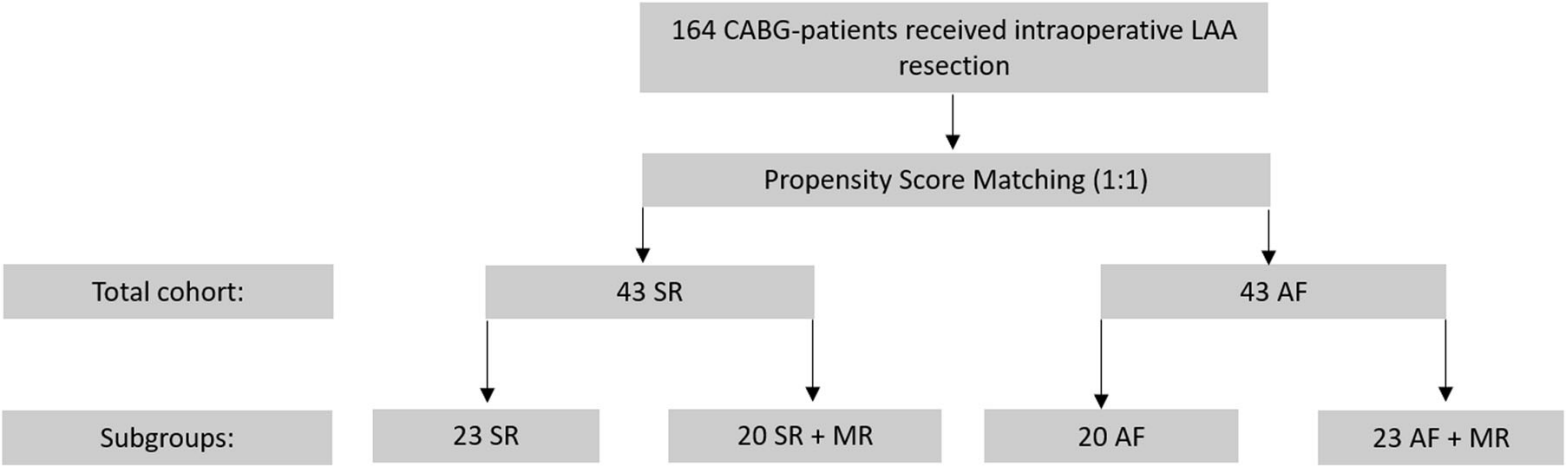
Flowchart of patient selection. A total of 164 CABG‐patients were propensity matched, resulting in a total cohort of 43 patients with AF and SR. Subgroup division was based on mitral valve regurgitation. AF, atrial fibrillation; CABG, coronary artery bypass graft; LAA, left atrial appendage; MR, mitral valve regurgitation; SR, sinus rhythm

A retrospective data analysis was performed in CABG patients undergoing LAA resection during bypass surgery from 2014 to 2018. The study was approved by the local ethics committee. The requirement for written informed consent was waived. Out of 164 patients, 50 patients without AF and 114 with a clinical history of AF were identified. AF and SR patients were propensity score (PS) matched, resulting in 2 groups of 43 subjects (Figure 1). Moreover, subgroups of MR were divided as follows: SR, SR + MR, AF and AF + MR. Follow‐up data on mortality, postoperative stroke and postoperative AF were collected between the years 2014 and 2021. Follow‐up data was obtained annually via postal or telephone questionnaire. In case of a missing response, the local registration office was contacted for mortality status.

### 2.2 Histology

**Figure 2:**
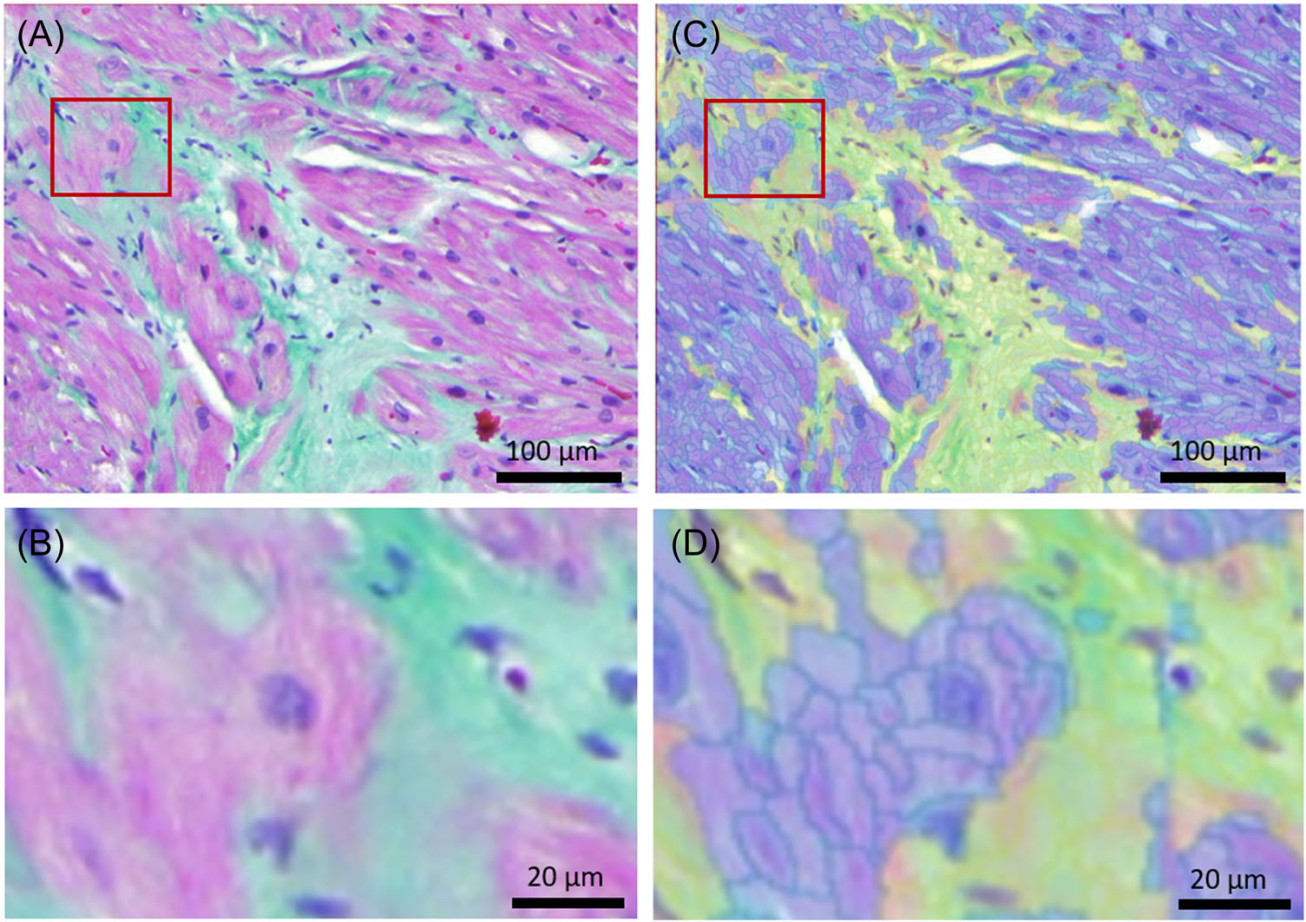
Digital segmentation of the left atrial appendage landscape. The Masson‐Goldner trichrome stained left atrial appendage (LAA) was segmented into superpixels via algorithm‐based classification. (A, B) present myocardiocytes (violet) and interstitial fibrosis (green). (C, D) exhibit identical tissue sections with algorithm‐detected interstitial fibrosis (lime‐green) and nonfibrotic areas (blue). (B, D) are selected magnified sections of (A) and (C) displaying the level of precision of the algorithm‐based segmentation

Histologically sectioned LAA specimens were stained with Masson‐Goldner trichrome using standard protocol. Each LAA specimen was represented by one complete histological slice. The stained LAA containing slides were scanned using a Mirax Desk histology scanner (Carl Zeiss MicroImaging GmbH) and converted into images using the Mirax Scan 1.12 software (3DHISTECH Ltd.). Image processing and bioimage analysis were enabled using the software platform QuPath 0.1.2.^14^ QuPath's DoG superpixel algorithm was utilized to divide the microscopic map of the LAA into pixel‐grouped segments called superpixels.^15^ The downsample factor was set to 2 and the Gaussian sigma to 2 µm. A random trees classifier was trained on 18 158 superpixels to detect fine strands of fibrosis (Figure 2). To limit quantification to myocardial interstitial fibrosis, endocardial, and epicardial fibrosis were selectively excluded during image processing.

### 2.3 Statistics

To raise the compatibility of both groups, a 1:1 PS matching was carried out using logistic regression. A prespecified compatibility distance termed caliper width of 0.2 standard deviation was applied. This width represents a fraction of a standard deviation of the logit for PS matching. A previous study presented caliper matching results superior when comparing to optimal and nearest neighbor matching regarding bias reduction.^16^, ^17^ As a result, two groups were formed, each entailing 43 patients with equal weighting of clinical parameters: age, CHA_2_DS_2_VASc score, gender, body mass index (BMI), arterial hypertension, hyperlipoproteinemia, smoker, diabetes mellitus, prior cardiac surgeries, MR, grade of MR, pacemaker, prior stroke, New York Heart Association (NYHA) class, number of coronary arteries affected, EuroSCORE II (Table S1).

IBM SPSS Statistics 24 (IBM Corp) was used for statistical analysis. Data were expressed as mean ± standard deviation for continuous variables. Normal distribution was assessed using the Kolmogorov–Smirnov Test. For the comparison of clinical parameter means of the SR and AF patients, a standardized mean difference was applied (SMD). A balanced distribution was considered for a SMD ≤ 10%. For the comparison of clinical parameters of the four subgroups, the nonparametric Kruskal–Wallis‐Test was utilized as normal distribution was found violated by the Shapiro–Wilk test. Statistical tests were exclusively carried out on propensity scored matched patient selection. Unpaired *t* tests were carried out for comparison of fibrotic remodeling. Sensitivity and specificity of LAA fibrosis in outcome prediction of patient mortality, postoperative stroke and postoperative AF was assessed using receiver operating characteristic analyses. Moreover, a Kaplan–Meier curve for survival distributions between subgroups was performed.

## 3 RESULTS

### 3.1 Patient baseline characteristics

PS matching of 86 Patients increased the compatibility (SMD < 10%) particularly for parameters CHA_2_DS_2_VASc score, the number of coronary arteries affected, males, arterial hypertension, hyperlipoproteinemia, diabetes mellitus, the number of prior cardiac surgeries, EuroSCORE II and prior stroke. A few imbalances (SMD > 10%) after PS matching remained for parameters BMI (SMD = 12.1%), Smoker (SMD = 21.7%), MR (SMD = 21.7%) and NYHA score (SMD = 12.7%), however, absolute differences remained comparatively small. The clinical parameters of all patients are summarized in Table S1.

### 3.2 LAA fibrosis of SR and AF patients

**Figure 3:**
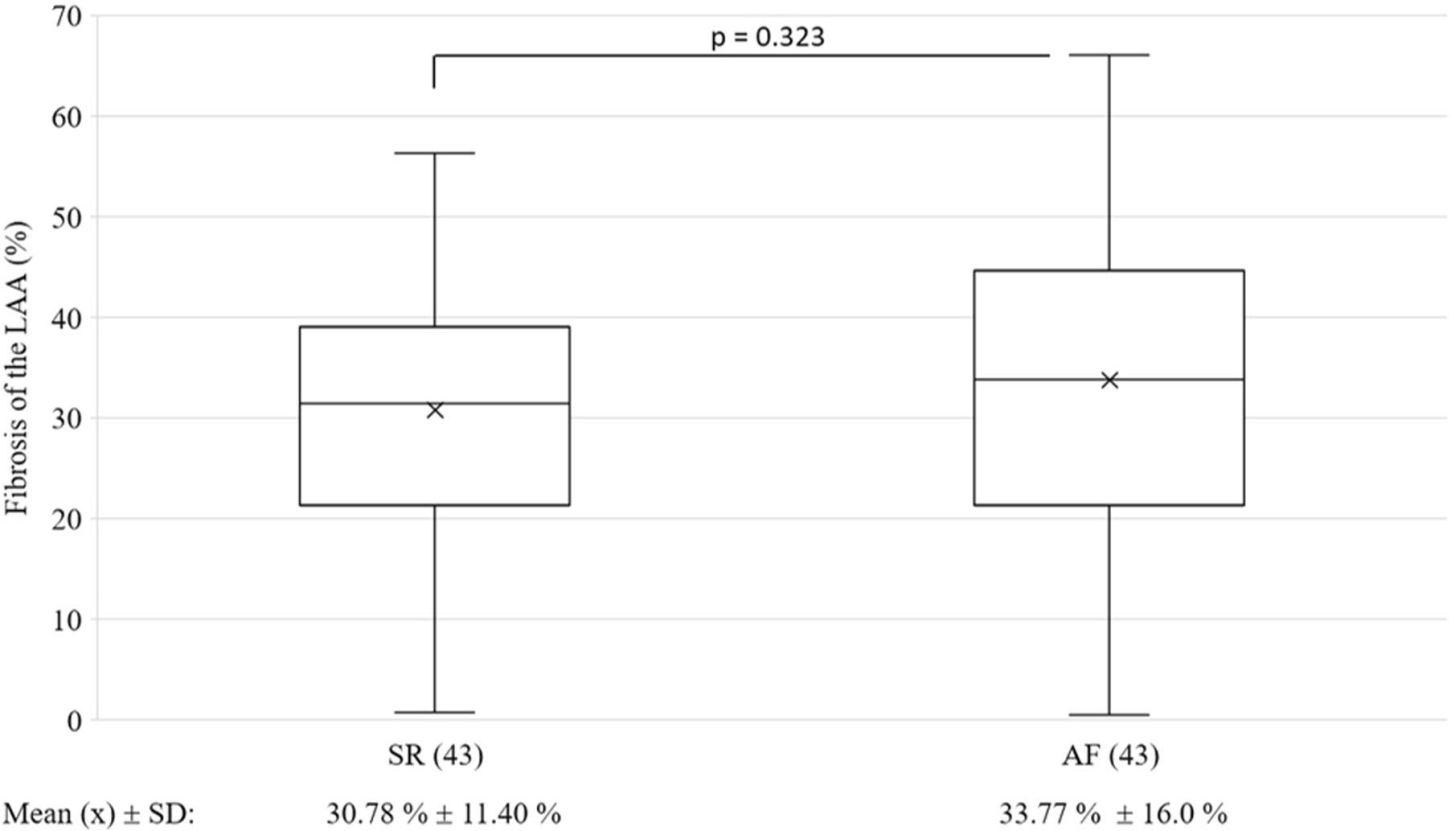
Interstitial fibrosis of the LAA in CABG patients with AF and SR. No significant difference in interstitial fibrosis was observed between the main propensity matched patient groups. Possibly, the underlying coronary heart disease, low to moderate grade mitral‐valve regurgitation and inclusion of paroxysmal AF reduce fibrotic contrast between both groups. Abbreviations: AF, atrial fibrillation; CABG, coronary artery bypass graft; LAA, left atrial appendage; SR, sinus rhythm

Comparison of interstitial fibrosis in the LAA between patients with SR and patients with AF (Figure 3), showed no significant difference (30.8 ± 11.4% and 33.8 ± 16.0%, respectively, *p* = .32). LAA fibrosis of SR and AF patients present no predictive (AUC < 0.7) value in determining mortality, postoperative stroke and postoperative AF (Table S3) as all tests qualify as “non‐useful.”

### 3.3 Subgroup analysis

**Figure 4:**
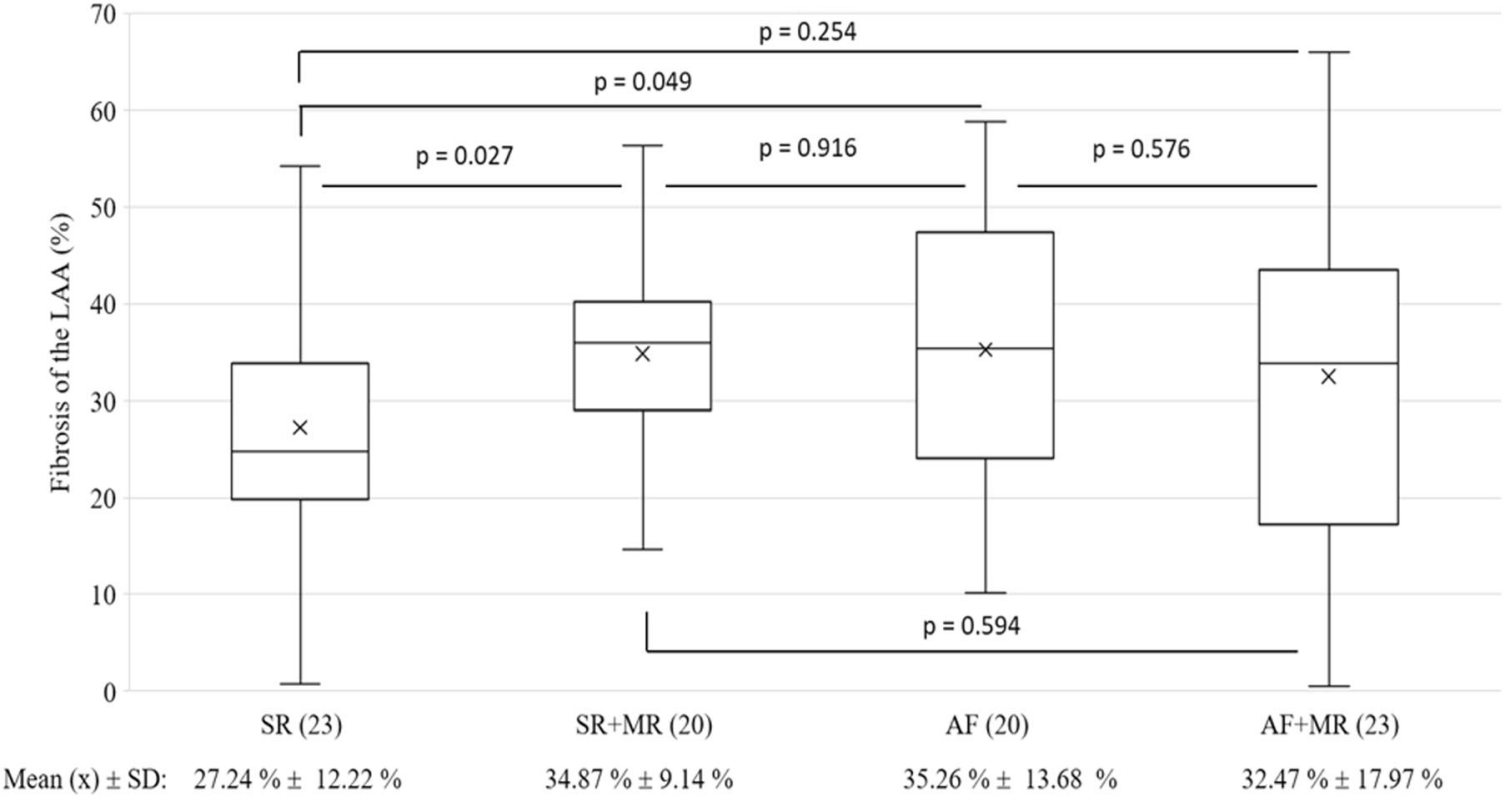
Interstitial fibrosis of the LAA in CABG patients with subgroups SR, SR + MR, AF, AF + MR. Consideration of mitral‐valve regurgitation showed LAA fibrosis for patients with SR + MR or AF was significantly increased compared to patients with SR. This implies LAA fibrosis may contribute as an arrhythmia substrate for AF but also in those with SR and coincidence of MR. AF, atrial fibrillation; CABG, coronary artery bypass graft; LAA, left atrial appendage; MR, mitral valve regurgitation; SR, sinus rhythm

**Figure 5:**
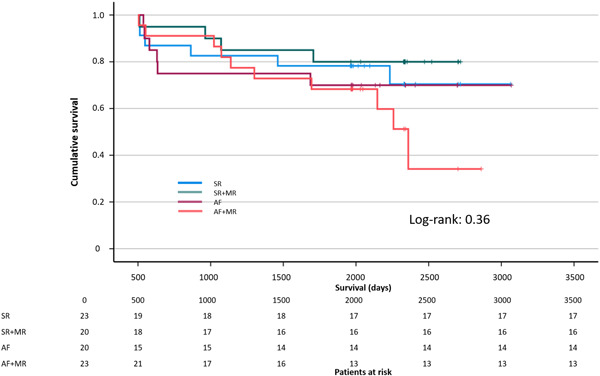
Kaplan–Meier curve of CABG subgroups: SR, SR + MR, AF, AF + MR. Regardless of SR, AF, or MR, our propensity matched CABG patients exhibited no significant differences in mortality. Anticoagulation therapy for AF patients and LAA resection as thromboembolic prophylaxis must be considered, presumably contributing to the similar survival distributions between CABG subgroups. AF, atrial fibrillation; CABG, coronary artery bypass graft; LAA, left atrial appendage; MR, mitral valve regurgitation; SR, sinus rhythm

The assessed clinical parameters presented homogeneity even after division into subgroups. The only statistically significant difference was the degree of MR. Clinical parameters of the subgroups are summarized in Table S2. Regarding patients with MR (Figure 4), a significant difference in interstitial fibrosis was found between patients with SR and patients with AF (27.2 ± 12.2% and 35.3 ± 13.7%, respectively, *p* = .049). Furthermore, comparison of patients with SR and patients with SR + MR presented significant fibrotic remodeling (27.2 ± 12.2% and 34.9 ± 9.1%, respectively, *p* = .027). No statistical differences in survival distributions (Figure 5) between all subgroups were identified (Log‐rank test = 0.36).

## 4 DISCUSSION

Recent therapeutic success of LAA isolation led to hypothesize that increased LAA fibrosis would act as a pathological substrate in patients with AF. Our study distinguishes itself from prior studies^2^, ^18^ in the usage of a self‐trained algorithm for quantification of LAA interstitial fibrosis, raising the objectivity of histological assessment. Furthermore, the present study is characterized by a relatively homogenous patient cohort attained via PS matching. The following observations were made throughout this study:

(i) In the total cohort, no statistical difference in LAA fibrosis for AF and SR patients was observed.
(ii) LAA fibrosis was not significantly associated with postoperative stroke, postoperative AF or overall mortality.
(iii) In the subgroups, patients with AF and patients with MR (MR + SR) had significantly increased LAA fibrosis compared to patients with SR.
(iv) Survival did not differ significantly between all subgroups following CABG.

### 4.1 LAA fibrosis of the total cohort

Comparison of the total cohort between patients with SR and AF showed no difference in LAA interstitial fibrosis. Both groups included patients with MR, which may have contributed towards lower fibrotic contrast. Moreover, greater fibrotic contrast would be expected when differentiating between paroxysmal, persistent and permanent AF, as observed previously.^19^ Additionally, the underlying coronary heart disease may have provided fibrotic alterations for both groups,^12^ diminishing the profibrotic effect of AF. Thus, fibrotic remodeling does not appear to differ among CABG patients with AF or SR.

Regarding follow up data for both groups, LAA fibrosis was not associated with postoperative stroke, postoperative AF and overall mortality. Previous studies have found LA fibrosis to be associated with increased stroke incidence.^20^, ^21^ We found that LAA fibrosis does not predict the risk of postoperative stroke. A possible explanation could be the surgical occlusion, which contributes towards the low stroke incidence in both patient groups, substantiated by the recent multicentre LAA occlusion study.^12^ Moreover, patients with AF receive long‐term if not life‐long anticoagulation therapy for thromboembolic prophylaxis, additionally lowering the risk of postoperative stroke.^22^ Furthermore, LAA interstitial fibrosis presented no prognostic value regarding postoperative AF for patients without clinical history of AF. Di Biase et al.,^11^ had observed that the ectopic foci triggering sites were only found in the LAA for 27% of AF patients. Additionally, fibrotic remodeling has been characterized as a structural remodeling process that usually occurs at later stages of AF disease progression, preceded by electromechanical alterations.^23^ Thus, LAA fibrosis may not serve as a useful predictor of AF among subjects without clinical history of AF or in early stages of disease.

### 4.2 LAA fibrosis in subgroups

Upon consideration of MR, our subgroup results suggest that increased LAA fibrosis may act as an arrhythmogenic substrate in patients with AF. This observation is supported by the histological study by Ma et al.,^24^ which additionally found increased synthesis of collagen I and III in the LAA of patients with AF compared to sinus rhythm. In patients with MR (MR + SR), significant fibrotic remodeling was observed compared to patients with SR. Our results correlate with previous histological studies of the posterior atrial wall.^25^ We find the hemodynamic alterations associated with MR to be capable of inducing significant fibrotic remodeling of the LAA, in absence of AF.

Our data do not support earlier findings that demonstrate a significant fibrotic difference between MR patients with and without AF (MR vs. MR + AF).^25^, ^26^, ^27^ In studies that showed significant differences, patients with persistent AF were often selected exclusively.^25^, ^26^ Furthermore, only patients with a low to moderate grade MR were included in this study. Patients with higher grade MR could present greater fibrotic remodeling in combination with AF.

### 4.3 The LAA as therapeutic target in AF treatment

Recent isolation studies of the LAA via catheter ablation, in addition to the standard procedure of pulmonary vein isolation, resulted in a significant reduction in AF recurrence rate in previous studies.^9^, ^10^, ^11^, ^28^, ^29^ The increased fibrotic remodeling in patients with AF observed in our subgroups, could imply that LAA fibrosis may contribute to induce AF. In patients with MR (SR + MR), increased LAA fibrosis could mark histological alterations preceding AF development. Our study implies why previous therapeutic success regarding LAA‐isolation may be associated with histological alterations.

### 4.4 Limitations

This retrospective study reduced absolute differences of clinical characteristics for the two patient groups AF and SR by carrying out 1:1 PS matching. However, PS matching only relates to the division of patients between SR or AF. The subsequent subgroups, although presenting a homogenous distribution of clinical characteristics (Table S2), are no longer subject to the original PS matching. Notably, regardless of whether PS matching was performed or not, retrospective studies generate hypotheses and cannot be used to represent causality. Moreover, a comparison to a healthy cardiac control group would have been desirable. Unfortunately, no postmortem LAAs of healthy controls were available.

## 5 CONCLUSION

Investigation of resected LAA during CABG surgery, presented no fibrotic difference between AF and SR patients. However, subgroups with AF or MR (MR + SR) showed significantly increased LAA fibrosis compared to patients with only SR. Thus, LAA fibrosis may contribute as an arrhythmia substrate for AF but also in those with SR and coincidence of MR. LAA fibrosis does not predict patient mortality, postoperative stroke or postoperative  AF.

## CONFLICT OF INTEREST

The authors declare no conflict of interest.

## Supporting information

Supporting information.

## Data Availability

The data that support the findings of this study are available on request from the corresponding author. The data are not publicly available due to restrictions as it is containing information that could compromise the privacy of research participants.
