# Supplementary material for: Impact of left atrial appendage fibrosis on atrial fibrillation in patients following coronary bypass surgery
Source: Clin Cardiol. 2022 Jul 21;45(10):1029–35. doi: 10.1002/clc.23884 (PMC9574744; doi:10.1002/clc.23884)
Supplement: Supplementary file 1 — Supporting information. [file CLC-45-1029-s001.docx]

**Supplement Data**

|  | SR  (original) | AF  (original) | SMD (%) before matching | SR  (matched) | AF  (matched) | SMD (%)  after matching |
| --- | --- | --- | --- | --- | --- | --- |
| Clinical Parameters | 50 | 114 |  | 43 | 43 |  |
| Age (years) *m* ± SD | 72.3 ± 7.8 | 73.1 ± 8.2 | 3.7 | 72.4 ± 9.1 | 71.5 ± 9.5 | 9.6 |
| CHA_2_DS_2_VASc Score *m* ± SD | 3.4 ± 1.4 | 3.2 ± 1.5 | 13.8 | 3.3 ± 1.4 | 3.4 ± 1.6 | 6.7 |
| Male *n* (%) | 40.0 (80.0) | 88.0 (77.2) | 10 | 35.0 (81.4) | 34.0 (79.1) | 7.9 |
| BMI (kg/m^2^) *m* ± SD | 28.9 ± 5.2 | 28.6 ± 5.2 | 3.8 | 29.1 ± 5.3 | 28.5 ± 4.7 | 12.1 |
| Arterial hypertension *n* (%) | 42.0 (84.0) | 101.0 (88.5) | 17.3 | 36.0 (83.7) | 36.0 (83.7) | 0.0 |
| Hyperlipoproteinemia *n* (%) | 47.0 (94.0) | 105.0 (92.1) | 9.9 | 41.0 (95.3) | 41.0 (95.3) | 0.0 |
| Smoker *n* (%) | 11.0 (22.0) | 36.0 (31.6) | 28.8 | 9.0 (20.9) | 12.0 (27.9) | 21.7 |
| Diabetes mellitus *n* (%) | 26.0 (52.0) | 40.0 (26.3) | 81 | 21.0 (48.8) | 20.0 (46.5) | 6.4 |
| Prior cardiac surgeries *n* (%) | 5.0 (10.0) | 3.0 (2.6) | 55.9 | 2.0 (4.7) | 2.0 (4.7) | 0.0 |
| MR *n* (%) | 24.0 (48.0) | 63.0 (55.3) | 20.6 | 20.0 (46.5) | 23.0 (53.5) | 19.7 |
| Grade of MR *m* ± SD | 1.7 ± 1.1 | 1.8 ± 0.9 | 10.1 | 1.7 ± 1.1 | 1.8 ± 0.9 | 10.0 |
| Pacemaker *n* (%) | 7.0 (14.0) | 17.0 (14.9) | 3.5 | 6.0 (13.7) | 5.0 (11.6) | 8.4 |
| Prior Stroke *n* (%) | 0.0 (0.0) | 4.0 (3.5) | 23.6 | 0.0 (0.0) | 0.0 (0.0) | 0.0 |
| NYHA Class *m* ± SD | 2.2 ± 0.9 | 2.2 ± 0.8 | 0.0 | 2.2 ± 0.9 | 2.3 ± 0.8 | 12.7 |
| No. of coronary arteries affected *m* ± SD | 2.9 ± 0.3 | 2.9 ± 0.4 | 30.3 | 2.9 ± 0.3 | 2.9 ± 0.3 | 9.7 |
| EuroSCORE II *m* ± SD | 4.0 ± 6.7 | 3.4 ± 5.1 | 10.1 | 3.9 ± 7.1 | 4.1 ± 5.8 | 3.1 |

Baseline parameters of patients with AF and SR before and after propensity score-matching

**Table S1.** Continuous variables: m ± SD: mean + standard deviation, categorical variables: n (%): number of events and corresponding percentage. Abbreviations: AF, atrial fibrillation; SR, sinus rhythm; SMD, standardized mean difference; COPD, chronic obstructive pulmonary disease; MR, mitral valve regurgitation; BMI, body mass index; NYHA, new york heart association.

|  | SR | SR+MR | AF | AF+MR | Kruskal-Wallis-Test |
| --- | --- | --- | --- | --- | --- |
| Clinical Parameters | 23 | 20 | 20 | 23 |  |
| Age (years) *m* ± SD | 70.9 ± 6.6 | 74.2 ± 8.9 | 69.8 ± 9.3 | 72.0 ± 9.1 | 0.53 |
| CHA_2_DS_2_VASc *m* ± SD | 3.1 ± 1.5 | 3.6 ± 1.4 | 3.2 ± 1.7 | 3.4 ± 1.5 | 0.24 |
| Male *n* (%) | 20.0 (87.0) | 15.0 (75.0) | 15.0 (75.0) | 19.0 (82.6) | 0.70 |
| BMI (kg/m^2^) *m* ± SD | 29.7 ± 5.8 | 28.2 ± 4.4 | 29.2 ± 4.5 | 27.9 ± 4.7 | 0.57 |
| Arterial hypertension *n* (%) | 19.0 (82.6) | 17 (85.0) | 18.0 (90.0) | 18.0 (78.3) | 0.77 |
| Hyperlipoproteinemia *n* (%) | 22.0 (95.7) | 19.0 (95.0) | 20.0 (100) | 21.0 (91.3) | 0.61 |
| Smoker *n* (%) | 6.0 (26.1) | 3.0 (15.0) | 7.0 (35.0) | 5.0 (21.7) | 0.52 |
| Diabetes mellitus *n* (%) | 10.0 (43.5) | 11.0 (55.0) | 8.0 (40.0) | 12.0 (52.2) | 0.74 |
| Prior cardiac surgeries *n* (%) | 0.0 (0.0) | 2.0 (10.0) | 1.0 (5.0) | 1.0 (4.4) | 0.50 |
| Grade of MR *m* ± SD | 1.0 ± 0.0 | 2.5 ± 2.0 | 1.0 ± 1.0 | 2.5 ± 1.0 | <0.001* |
| Pacemaker *n* (%) | 2.0 (8.7) | 4.0 (20.0) | 3.0 (15.0) | 2.0 (8.7) | 0.64 |
| NYHA Class *m* ± SD | 2.0 ± 1.0 | 2.4 ± 0.8 | 2.2 ± 0.8 | 2.2 ± 0.9 | 0.24 |
| No. of coronary arteries affected *m* ± SD | 2.9 ± 0.2 | 2.9 ± 0.3 | 2.9 ± 0.3 | 2.9 ± 0.3 | 0.68 |
| EuroSCORE II *m* ± SD | 2.1 ± 2.8 | 6.0 ± 9.5 | 2.3 ± 3.7 | 5.7 ± 6.6 | 0.08 |

Baseline parameters of all CABG subgroups

**Table S2.** Continuous variables: m ± SD: mean + standard deviation, categorical variables: n (%): number of events and corresponding percentage, *: significant difference between groups. Abbreviations: SMD, standardized mean difference; COPD, chronic obstructive pulmonary disease; MR, mitral valve regurgitation; BMI, body mass index; NYHA, new york heart association.

ROC Analyses of LAA fibrosis for mortality, postoperative stroke and postoperative AF for the propensity matched cohort

|  | | Events | | Fibrosis Cut-Off value (%) | AUC | Significance | 95% CI | | | Quality |
| --- | --- | --- | --- | --- | --- | --- | --- | --- | --- | --- |
|  | |  |  | |  |  | Lower bound | | Upper bound |  |
|  | SR Patients (total 43) | | | | | | | | | |
| Mortality | | 10 | 39.8 | | 0.45 | 0.66 | 0.24 | 0.67 | | nonuseful |
| Postoperative Stroke | | 0 | - | | - | - | - | - | | nonuseful |
| Postoperative AF^a^ | | 6 | 38.2 | | 0.39 | 0.40 | 0.12 | 0.67 | | nonuseful |
|  | AF Patients (total 43) | | | | | | | | | |
| Mortality | | 16 | 42.8 | | 0.53 | 0.70 | 0.32 | 0.75 | | nonuseful |
| Postoperative Stroke | | 3 | 56.1 | | 0.67 | 0.32 | 0.23 | 1.00 | | nonuseful |

**Table S3.** ^a^Patients without clinical history of AF. ROC – receiver operating characteristic, AUC – area under the curve, AF- atrial fibrillation, CI – confidence interval
